# Smell and 3D Haptic Representation: A Common Pathway to Understand Brain Dynamics in a Cross-Modal Task. A Pilot OERP and fNIRS Study

**DOI:** 10.3389/fnbeh.2019.00226

**Published:** 2019-09-26

**Authors:** Sara Invitto, Roberta Montinaro, Vincenzo Ciccarese, Irene Venturella, Giulia Fronda, Michela Balconi

**Affiliations:** ^1^Human Anatomy and Neuroscience Laboratory, Department of Biological and Environmental Sciences and Technologies, University of Salento, Lecce, Italy; ^2^Laboratory of Interdisciplinary Research Applied to Medicine, University of Salento-Vito Fazzi Hospital, Lecce, Italy; ^3^Istituto Santa Chiara, Rome, Italy; ^4^Research Unit in Affective and Social Neuroscience, Department of Psychology, Catholic University of Milan, Milan, Italy

**Keywords:** OERPs, olfactory perception, haptic perception, fNIRS, delta rhythm, cross-modal perception

## Abstract

Cross-modal perception allows olfactory information to integrate with other sensory modalities. Olfactory representations are processed by multisensory cortical pathways, where the aspects related to the haptic sensations are integrated. This complex reality allows the development of an integrated perception, where olfactory aspects compete with haptic and/or trigeminal activations. It is assumed that this integration involves both perceptive electrophysiological and metabolic/hemodynamic aspects, but there are no studies evaluating these activations in parallel. The aim of this study was to investigate brain dynamics during a cross-modal olfactory and haptic attention task, preceded by an exploratory session. The assessment of cross-modal dynamics was conducted through simultaneous electroencephalography (EEG) and functional near-infrared spectroscopy (fNIRS) recording, evaluating both electrophysiological and hemodynamic activities. The study consisted of two experimental sessions and was conducted with a sample of ten healthy subjects (mean age 25 ± 5.2 years). In Session 1, the subjects were trained to manipulate 3D haptic models (HC) and to smell different scents (SC). In Session 2, the subjects were tested during an attentive olfactory task, in order to investigate the olfactory event-related potentials (OERP) N1 and late positive component (LPC), and EEG rhythms associated with fNIRS components (oxy-Hb and deoxy-Hb). The main results of this study highlighted, in Task 1, a higher fNIRS oxy-Hb response during SC and a positive correlation with the delta rhythm in the central and parietal EEG region of interest. In Session 2, the N1 OERP highlighted a greater amplitude in SC. A negative correlation was found in HC for the deoxy-Hb parietal with frontal and central N1, and for the oxy-Hb frontal with N1 in the frontal, central and parietal regions of interests (ROIs). A negative correlation was found in parietal LPC amplitude with central deoxy-Hb. The data suggest that cross-modal valence modifies the attentional olfactory response and that the dorsal cortical/metabolic pathways are involved in these responses. This can be considered as an important starting point for understanding integrated cognition, as the subject could perceive in an ecological context.

## Introduction

Cross-modal perception allows the olfactory pathway to integrate with other sensory modalities (Hanson-Vaux et al., 2013; Leleu et al., 2015a, b). The olfactory representations are processed by multisensory cortical pathways, where the aspects related to haptic sensations are integrated. This complex reality allows the development of an integrated perception, where the olfactory aspects compete with haptic and/or trigeminal activations. It is assumed that this integration involves both perceptive electrophysiological and metabolic/hemodynamic aspects, but there are no studies evaluating these activations in parallel (Shepherd, 2006).

Moreover, olfactive cognition (i.e., food odor) is modulated by haptic perception in young subjects and by visual perception in geriatric subjects, so this interaction seems to be age-related (Merkonidis et al., 2015). Recent research investigated the cross-modal association between taste and vision, highlighting a correlation between shape and pleasantness (Ngo et al., 2011; Hanson-Vaux et al., 2013; Kaeppler, 2018).

Although, interesting research topics exist on cross-modal interactions, multi-sensorial interactions between olfaction and haptic manipulation have not been sufficiently investigated. Previous work investigated how the P3 ERP can be differently modulated in a visual recognition task when the stimulus was processed through an olfactory and haptic cross-modal pathway (Invitto et al., 2019). Following on from previous results, we can consider that olfactory and haptic cross-modal interaction could be localized in the left hemisphere, particularly in the occipital–temporal–parietal stream. This topographic localization can be identified as the dorsal pathway, linked to stimulus localization (Goodale et al., 2005; Bornkessel-Schlesewsky and Schlesewsky, 2013; Kuang and Zhang, 2014; Invitto et al., 2019).

Studies focusing on ERPs and electroencephalographic rhythms report slower frequencies in olfactory and haptic perceptive tasks, in particular delta and theta for the olfactory system and mu for the haptic system during motor action (Martin, 1998; Freyer et al., 2012; Rihm et al., 2014; Riečanský et al., 2015). Studies investigating the functional near infrared spectroscopy (fNIRS) and the olfactory system indicate that the deoxy-hemoglobin (deoxy-Hb) level did not change with a bilateral increase of oxy-hemoglobin (oxy-Hb) (Ishimaru et al., 2004), while there is an involvement of the orbitofrontal cortex (Kokan et al., 2011).

The purpose of this study was to describe and examine brain dynamics during a cross-modal olfactory and haptic attention task, preceded by an evaluation and exploratory session. The assessment of cross-modal dynamics was conducted through electroencephalography (EEG) and fNIRS, in order to evaluate the electrophysiological and hemodynamic responses. Specifically, the variations of the delta (Harada et al., 1998; Piarulli et al., 2018) and the mu rhythms (McFarland et al., 2000; Peled-Avron et al., 2016) were considered in the first exploratory training session and were correlated with the oxy-Hb and deoxy-Hb variations recorded by fNIRS. In Session 2, the olfactory event-related potentials (OERP) N1 and late positive component (LPC), specifically the olfactory response during an attentive task (Pause et al., 1996), were investigated. Discriminating whether cross-modal valence modifies the attentional olfactory response and what the cortical/metabolic pathways involved in these responses are can be an important starting point for understanding integrated cognition, as the subject could perceive in an ecological setting (Neisser, 1988).

## Materials and Methods

### Subjects

Ten female university students (mean age: 25 ± 5.2 years) were recruited for the experiment. The exclusion criteria included having a prior history of neurological or psychiatric illness, or current or previous psychoactive medication use. None of the recruited subjects belonged to this category. All participants were informed about the experimental procedure and gave written consent prior to the experiment. The research was conducted according to the Helsinki declaration and was approved by the Ethics Committee of the Psychology Department, Catholic University of Milan.

### Experimental Sessions

**FIGURE 1:**
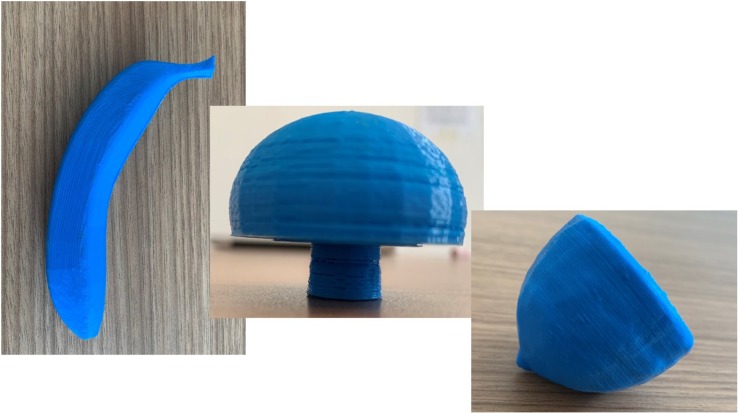
Examples of three-dimensional haptic shapes printed for the experiment.

In Session 1, the subject breathed the odorants, some of which were also presented in the haptic mode (see Table 1). The shapes, haptic 3D models (printed using the 3D Blender 2.74 platform), were all monochromatic (blue) and of the same consistency (rigid plastic) to avoid bias due to further stimulation variability (see Figure 1).

**TABLE 1:** Name of the chemical odorants used, the perception linked to the chemical odorants and the condition (i.e., smell or cross-modal haptic smell) of the administration during the task in Session 1.

**Name of the odorant**	**Perception**	**Condition**
Cinnamaldehyde	Cinnamon	Cross-modal
Citral	Lemon	Cross-modal
Hexanal	Grass	Smell
Phenethyl alcohol – PEA	Rose	Smell
Carvone	Mint	Smell
1-Octen-3-ol	Mushroom	Cross-modal
Isoamyl acetate	Banana	Cross-modal
4-Ethylphenol	Flower/solvent	Smell

The order of presentation of the odorants was pseudo-randomized.

The haptic session was balanced by different forms just as the smells administrated. The haptic cross-modal session was focused on four different shapes: a rounded shape (lemon), an elongated and rolled shape (cinnamon), a shape consisting of a rounded part and an elongated part (the mushroom), and an elongated and curved shape (banana). Different forms elicit different sympathetic or parasympathetic activations and are associated with pleasant (round shapes) or less pleasant (pointed shapes) perceptions (Ngo et al., 2011; Hanson-Vaux et al., 2013). In order to avoid an emotional attribution bias dependent form, we chose to balance the forms presented by choosing different shapes.

Olfactive stimulations were administrated in a blind modality via a Plexiglas tube connected to an olfactometer (Invitto et al., 2018). The subject was given no visual information about the odorants or the 3D shapes. During the stimulation phase the subject breathed the odor in the olfactory condition or manipulated the shape while perceiving the odor (cross-modal condition). Each stimulation had a duration of 60 s during which the subject was in a resting state.

After each odorant presentation, a Visual Analogic Scale was administrated to the subjects to investigate the pleasantness, the level of arousal and the familiarity of the olfactive stimulus. Session 1 had a duration of about 20 min.

Session 2 consisted in a Go/No-Go task, with an interstimulus interval of 15 s and a stimulus presentation of 350 ms (S1 = 15 Stimulations; S2 = 15 Stimulations – Ratio S1–S2 = 50%).

During the Go/No-Go task session, the subjects were asked to press a computer key on the left-hand side of the keyboard if the stimulus recognition was encoded through the olfactory modality and a computer key on the right-hand side of the keyboard if the stimulus recognition was encoded in the cross-modal way. Session 2 lasted about 30 min.

### EEG–fNIRS Recording

For the EEG signal recording, a 16-channel portable EEG system (V-AMP: Brain Products, Munich) was used with the following electrode placements: Fp1, Fp2, F7, Fz, F8, C3, Cz, C4, TP7, TP8, P3, Pz, P4, O1, and O2. The electrodes were placed on individuals’ scalps using a cap that allows combined EEG electrode and fNIRS optode placement (actiCAP, Brain Products). Specifically, EEG electrodes were used to record EEGs from the active scalp sites referring to the earlobes (international system 10/5, Oostenveld and Praamstra, 2001). Data acquisition took place with a sampling frequency of 500 Hz and with a frequency band of 0.01–40 Hz. A common offline average reference was used to limit the problems associated with signal-to-noise ratio (Ludwig et al., 2009). Furthermore, an EOG electrode was placed on the external canthi. The impedance of each electrode was monitored before data collection and was kept below 5 kΩ and stored for offline averaging. To reduce high frequency noise, the signal was low pass filtered at 30 Hz (slope 24 dB/octave). Removal of eye-movement artifacts was performed using ocular correction with independent component analysis. Then, to exclude external rumor and artifacts provided by body movements, artifact rejection was performed to discard epochs contaminated by artifacts or other signals exceeding the amplitude threshold of ± 110 μV. The EEG data were subsequently analyzed in the follow frequency bands, identified after a fast Fourier transform (FFT): delta (0.5–4 Hz), theta (4–7.5 Hz), alpha/mu (7.5–12.5 Hz), and beta (12.5–20 Hz) (Keil et al., 2003). The filtered signal (epoch 1000 ms) samples were squared to avoid a signal proportional to the power of the EEG frequency band.

For each EEG channel, a calculation of the average individual power value was performed for each experimental condition and the basic recordings.

The F7–F8, C3–C4, P3–P4 electrode couples were used for the statistical analysis. Three regions of interests (ROIs) were then calculated as follows: frontal (F7–F8), central (C3–C4) and parietal (P3–P4).

### fNIRS Recording and Signal Processing

For the recording of hemodynamic responses, a NIRScout system (NIRx Medical Technologies LLC, Los Angeles, CA, United States) was used. A 16-optode matrix (eight injectors and eight detectors) was placed on the prefrontal and centro-parietal regions according to the international 10/5 system. The optodes were placed 3 cm from each other through the use of a cap for the combined EEG and fNIRS montage. A near-infrared light at two wavelengths (760 and 850 nm) was used. The injectors were located over the positions: F5–F6, FCC3h–FCC4h, CCP5h–CCP6h, P1–P2, while the detectors were placed at the following positions: F3–F4, FCC5h–FCC6h, CCP3h–CCP4h, PO3–PO4. The following channels were acquired: Ch1 (F5–F3), Ch2 (FCC3h–FCC5h), Ch3 (FCC3h–CCP3h), Ch4 (CCP5h–FCC5h), Ch5 (CCP5h–CCP3h), Ch6 (P1–PO3), Ch7 (F6–F4), Ch8 (FCC4h–FCC6h), Ch9 (FCC4h–CCP4h), Ch10 (CCP6h–FCC6h), Ch11 (CCP6h–CCP4h), Ch12 (P2–PO4).

Variations in the concentration of oxygenated (O_2_Hb) and deoxygenated (HHb) hemoglobin were continuously recorded after the acquisition of a preliminary baseline of 120 s. A sampling frequency of 6.25 Hz was used to record the signal from the 12 NIRS channels. NirsLAB software (v2014.05, NIRx Medical Technologies LLC, Glen Head, NY, United States) was used to analyze the signal based on its wavelength and position, which led to values for changes in the concentration of oxygenated and deoxygenated hemoglobin for each channel. Even if the fNIRS signal is not affected by movement artifacts if compared to fMRI (Hucke et al., 2018), the raw O_2_Hb and HHb data for each channel were digitally filtered to a filtered band at 0.01–0.3 Hz to exclude artifacts provided by body movements.

In Session 1, the mean concentration of O_2_Hb and HHb for each channel was calculated by averaging the data. The averaging was obtained across the eight stimuli segmented by the presentation mode (smell vs. cross-modal).

In Session 2, the mean concentration of O_2_Hb and HHb for each channel was calculated by averaging the data related to the two mode stimuli presentation (smell vs. cross-modal), 6 s after the onset of each stimulus.

For each channel and subject, the mean concentrations in the time series were considered. The effect size in every block was calculated as the difference of the means of the block (*m*2) and the baseline (*m*1) divided by the standard deviation (SD) of the baseline: *d* = (*m*2 – *m*1)/SD (Cohen’s *d* value). The procedure was applied for both O_2_Hb and HHb variations. This normalized index was averaged regardless of the unit since the effect size was not affected by the differential path length factor, which overcame the fact that fNIRS raw data were originally relative values and could not be directly compared across subjects or channels (Schroeter et al., 2003; Matsuda and Hiraki, 2006; Shimada and Hiraki, 2006).

To compare EEG data and fNIRS data, the following ROIs were created: F (F5–F3; F6–F4), C (FCC3h–CCP3h; FFC4h–CCP4h), P (P1–PO3; P2–PO4).

### Statistical Analysis and Results

#### Session 1

##### EEG FFT

Non-parametric testing (Friedman and Wilcoxon’s tests) was performed due to the sample size.

In Task 1, significant differences in alpha/mu and delta (Friedman test 0.000) were found in the frontal, central and parietal ROIs, both in the cross-modal and in the smell conditions. In all the ROIs, delta waves were more prominent (see Table 2).

**TABLE 2:** Friedman ranks.

**ROI**	**Condition**	**Alpha–mu**	**Delta**
F	Smell	3.22	4.89
F	Cross-modal	2.89	5
C	Smell	3.56	4.89
C	Cross-modal	3.11	5
P	Smell	3.78	4.56
P	Cross-modal	3.67	4.78

*Significance (Friedman test 0.000): frequency band mean power.*

No significant differences between conditions were found in alpha (frontal *Z* = −0.770, *p* = 0.441; central *Z* = −1.244, *p* = 0.214; and parietal *Z* = 1.2, *p* = 0.21) and in delta (frontal *Z* = −0.700, *p* = 0.484; central *Z* = 0.00; *p* = 0.100; and parietal *Z* = −1.4, *p* = 0.161).

Furthermore, as can be seen in Table 2, there is a small trend that shows a greater frequency band mean power on alpha/mu in the smell condition and delta in the cross-modal condition.

As a control, the same analysis was performed in the resting state condition and recorded before Task 1. The analysis confirmed a greater proportion of delta vs. alpha/mu (frontal = alpha 0.036 vs. delta 1.33; central = alpha 0.079 vs. delta 1.152; and parietal = alpha 0.127 vs. delta 2.307). Subtracting the resting state condition (the first 60 s of the EEG recording), we found the same proportion observed during the task. The subtracted values show non-significant differences both in alpha and delta (alpha: frontal *Z* = −0.652, *p* = 0.515; central *Z* = −0.338, *p* = 0.735; parietal *Z* = −1.352, *p* = 0.176 and delta: frontal *Z* = −1.53, *p* = 0.249; central *Z* = −0.943, *p* = 0.345; parietal *Z* = −1.753, *p* = 0.080).

##### fNIRS

**FIGURE 2:**
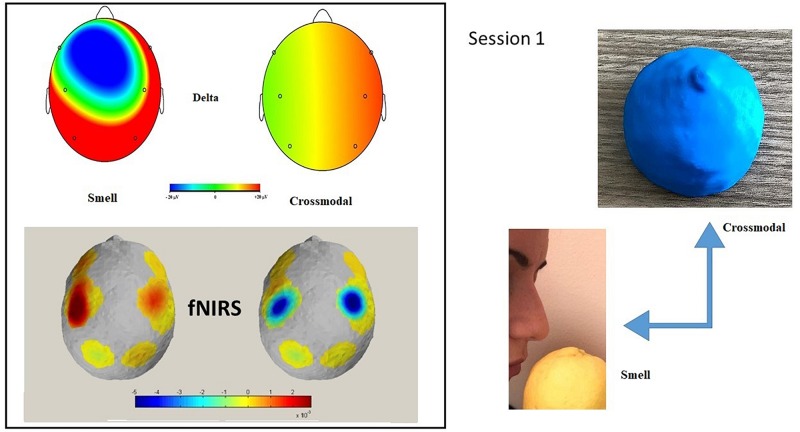
Session 1: Topographic distribution of the delta EEG power and fNIRS activation in smell and cross-modal conditions during Session 1. fNIRS mapping indicates an increase of O_2_Hb activity in central and parietal areas in the smell condition compared to the cross-modal condition. In the smell condition, a significant positive correlation was found in delta power in the frontal and central Oxy. No correlation was found in cross-modal condition. The maps are based on the 16 EEG and fNIRS derivations according to the international 10/5 system.

Wilcoxon’s test (paired) showed significant differences in oxy-Hb sensors in the central (*Z* = −2.191; *p* = 0.028) and in the parietal ROI (*Z* = −2.090; *p* = 0.037), in terms of greater activation in the smell condition (see Table 3 and Figure 2).

**TABLE 3:** Oxy sensor mean and standard error.

**Condition**	**Oxy-ROI**	**Mean**	**SEM**
Smell	F	0.179	0.60
	C	0.1676	0.28
	P	0.108	0.13
Cross-modal	F	0.067	0.31
	C	–0.1187	0.33
	P	–0.021	0.27

No significant differences were found in deoxy-Hb measures (frontal *Z* = −1.172; *p* = 0.241; central *Z* = −0.663; *p* = 0.508; and parietal *Z* = −1.274; *p* = 0.203).

##### Correlation measure

For non-parametric correlation analysis, Spearman’s coefficient was performed, a non-parametric correlation R index on continuous variables.

No significant correlation was found in the cross-modal condition between alpha/mu and delta spectral power and the concentration of oxygenated and deoxygenated hemoglobin. In the smell condition, a significant correlation was found between alpha/mu and in delta power and the frontal oxy and central oxy with central and parietal EEG ROI and in frontal deoxy with frontal EEG ROI (sign = 0.050; *R* = 0.667) (see Table 4 and Figure 2).

**TABLE 4:** The main significant correlations between NIRS and EEG values during co-registration in the smell condition.

**Rhythms**	**NIRS**		**Frontal EEG ROI**	**Central EEG ROI**	**Parietal EEG ROI**
Delta	Frontal oxy	Sign	0.170	0.030	0.036
		*R*	0.500	0.717	0.700
	Central oxy	Sign	0.350	0.007	0.005
		*R*	–0.356	0.817	0.833
Alpha/mu	Frontal deoxy	Sign	0.050	0.576	0.637
		*R*	0.667	–0.217	–0.183

To better understand the effect of the non-parametric analysis, a multifactorial analysis repeated the measure with a 2 × 3 × 2 design, with ROI (frontal, central, and parietal), and with condition (smell and cross-modal modality) and rhythms (alpha and delta) as factors that were performed. The null hypothesis has been verified through the Mauchly sphericity test. The sphericity of the data can be assumed both for the condition and the rhythm factors, not for the ROI factor (W of Mauchly = 0.166; *p* = 0.002).

The results of alpha analysis showed a non-significant value for the condition (*F* = 4.14; *p* = 0.065), non-significant values for the ROIs (*F* = 1.17; *p* = 0.334) and a significant value for rhythms (*F* = 5.423, *p* = 0.048) in the direction of a greater presence of delta (delta = 1.04 vs. alpha 0.66).

In Session 1, the same analysis was carried out with fNIRS values with a multifactorial model with repeated measurements using a 2 × 3 × 2 design with condition (smell vs. cross-modal), ROI (frontal, central and parietal) and fNIRS (oxy and deoxy). No significant results were found.

#### Session 2

##### OERP and FFT analysis

N1 OERP, analyzed with a Wilcoxon test (paired), displayed a greater amplitude in the parietal ROI for the smell condition (*Z* = −2.073; *p* = 0.038; smell amplitude −5.73 μV vs. cross-modal amplitude 2.75 μV). No significant difference was found in latency. LPC did not highlight significant differences in latency or in amplitude values. Single non-parametric analyses on alpha/mu rhythms and delta, between the two conditions (smell vs. cross-modal), did not highlight differences in frontal, central and parietal ROI.

##### NIRS

**FIGURE 3:**
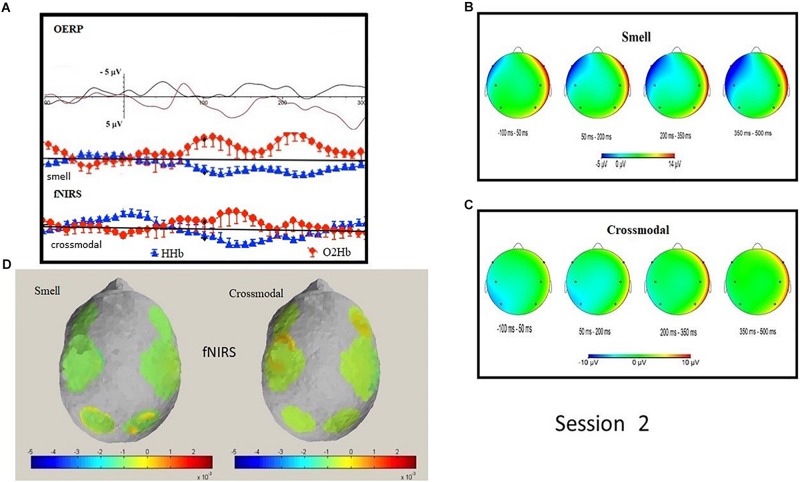
Session 2: Grand average OERP waveforms comparison and O_2_Hb and HHb signal in smell and cross-modal conditions **(A)**; topographic mapping of OERP amplitude in four different temporal ranges in smell **(B)** and cross-modal conditions **(C)**; topographic representation of fNIRS O_2_Hb and HHb activity in smell and cross-modal conditions **(D)**.

A Wilcoxon test (paired) did not highlight significant oxy and deoxy differences between cross-modal and smell conditions. Instead, significant negative correlations were found in the cross-modal condition in deoxy parietal with frontal and central N1 OERP amplitude and oxy frontal with N1 amplitude in frontal, central and parietal ROIs (see Table 5 and Figure 3). Furthermore, a negative correlation was found in LPC parietal amplitude with deoxy central (sign = 0.050; *R* = −0.632).

**TABLE 5:** The correlations between N1 and fNIRS values in Session 2.

**Cross-modal condition**		**N1 frontal**	**N1 central**	**N1 parietal**
Deoxy frontal	Sign	0.511	0.260	0.117
	*R*	0.236	0.394	0.527
Deoxy central	Sign	0.676	0.676	0.511
	*R*	–0.152	–0.152	0.511
Deoxy parietal	Sign	0.009	0.029	0.138
	*R*	–0.770	–0.685	–0.503
Oxy frontal	Sign	0.006	0.023	0.011
	*R*	–0.794	–0.745	–0.758
Oxy central	Sign	0.385	0.162	0.128
	*R*	0.309	0.479	0.515
Oxy parietal	Sign	0.777	0.987	0.603
	*R*	–0.103	0.006	–0.188

As in the Session 1, in Session 2 a repeated measure general linear model analysis was performed on EEG, with a 3 × 2 × 2 design, considering ROI (frontal, central, and parietal), condition (smell and cross-modal), and rhythms (alpha and delta) as factors. Mauchly’s sphericity test yielded significant values for ROI (Mauchly’s = 0.174; *p* = 0.001), condition × rhythms (1.87; *p* = 0.001) and ROI × rhythms (W of Mauchly = 0.178; *p* = 0.001). The analysis did not yield any significant results.

In Session 2, the same analysis was performed with the fNIRS values with a 2 × 3 × 2 design considering condition (smell × cross-modal), ROI (frontal, central and parietal) and fNIRS (oxy and deoxy). The Mauchly sphericity test yielded significant values for the interaction condition × ROI (W of Mauchly = 0.176; *p* = 0.002). The analysis carried out did not yield significant differences except in the interaction of condition for ROI (*F* = 13.22; *p* = 0.007) in the direction of more positive values in the smell condition (frontal = 0.011; central = 0.016; and parietal = 0.144) vs. more negative values in the cross-modal condition (frontal = −0.085; central = 0.056; and parietal = −0.097).

These results would suggest that performing a multivariate model did not improve the evidence with regards to the previous analysis. This is probably due to the limited sample size.

## Discussion and Conclusion

The olfactory representation is, unlike visual and auditory representation, a perception where the stimulus is processed and easily stored in the memory (for example, in the literature there is little information about olfactory working memory) (Dade et al., 2001; Yeshurun et al., 2008; Jönsson et al., 2011; Lenk et al., 2014). Olfactory perception always appears to be linked to phonological or “imagery” semantic aspects (Jönsson et al., 2011). We can also deduce from recent research findings that in all cases there is a strong connection, at least as shared pathways, between the olfactory system and visual and spatial representation (Schaefer and Margrie, 2007; Stettler and Axel, 2009). Furthermore, a study with positron emission tomography illustrated how both olfactory and face working memory engaged the dorsolateral and ventrolateral frontal cortex; a conjunction analysis indicated overlap in the distribution of parietal activity in the two sensory models (Dade et al., 2001). The findings support the idea that olfactory working memory processes engage frontal cortical areas independent of the modality of input, but do not rule out the possibility of modality-specific neural populations within the dorsolateral or ventrolateral cortex (Dade et al., 2001; Invitto et al., 2019). In this work, we have therefore tried to analyze the representation of these two aspects by comparing an aspect of pure olfactory stimulation with an aspect linked to the olfactory stimulation connected to an active haptic stimulation, using the 3D shapes associated to the perceived smell. The level of cortical activation was evaluated both through electrophysiological parameters, particularly through OERP, and through a slower hemodynamic response, observed through an fNIRS co-registration. The first main result of this study showed significant differences both in EEG and in fNIRS recordings in the first task, with olfactory and cross-modal stimulation. The fNIRS oxy-Hb showed a greater response during the smell condition in the central and parietal ROIs in parallel with an increase in activation of EEG delta rhythm, particularly linked to olfactive perception, in frontal, central and parietal ROIs. This is in line with the literature which points to a greater presence of slow rhythms, in particular delta, linked to olfactory stimulation (Schriever et al., 2017). Indeed, in Session 1 we found a more evident paired trend of both EEG and hemodynamic modulations, based on the smell condition.

The functional significance of delta oscillations in cognitive processing in central and parietal ROIs is strictly linked to perceptual, sensory-motor and cognitive processing (Harmony, 2013) and is present in the Go/No-Go task. Moreover, delta waves are related to the inhibition of non-relevant stimuli and in signal matching and decision making (Başar et al., 2000; Harmony, 2013).

In Session 2, the N1 OERP resulted in a greater amplitude smell condition, particularly in the frontal area. A negative correlation was found in HC for deoxy-Hb parietal with frontal and central N1.

This result mainly highlights the significant increase of frontal and central N1 in the cross-modal condition for electrophysiological measures (higher peak amplitude) in concomitance with a significant increase of activity for the parietal brain areas (observed through a negative correlation with deoxy-Hb). That is, the brain network supporting HC is underlined, by both EEG and fNIRS measures, as a form of focused activation which includes a larger brain network. We can hypothesize a ventral–dorsal stream (Binkofski et al., 1999), following other research findings, also for olfactory/cross-modal processes, and not only for visual/olfactory processes (Kuang and Zhang, 2014). Here, it seems that the dorsal and ventral pathways are predominantly a meta-function linked to the identification of the spatial representation, the form, its representation and its meaning (Marois et al., 2000; Zachariou et al., 2014).

Furthermore, a significant correlation was observed between N1 (i.e., frontal, central, and parietal localization) with the oxy-Hb frontal. This effect is a significant marker of hemodynamic and metabolic activity related to a specific electrophysiological trend: when the specific ROI shows a significant increased (or decreased) activity, the hemodynamic measure demonstrates a concomitant trend of increasing/decreasing activity. Following this, we may suppose that a primary sensorial activation (i.e., N1), however, evident in the three main locations, can be seen in a metabolic aspect in frontal oxy-Hb. It is particularly interesting how the literature refers to the activation of frontal aspects in oxy-Hb as both related to emotional aspects (negatively correlated to depression) (Kawano et al., 2016) and to subcortical activations (Sakatani et al., 1999). Considering that this sensory activation registered with OERPs is greater for the olfactory condition, we can hypothesize that specifically olfactory sensory stimulation involves subcortical and emotional processes, evident at the neuroimaging level. Indeed, the line of research that sees olfactory impairment as a biomarker also in depression (Pause et al., 2001; Atanasova et al., 2008; Croy and Hummel, 2017) would serve as a theoretical confirmation of the involvement of the olfactory aspects in emotional components.

A negative correlation was found in amplitude of LPC parietal with deoxy-Hb central. This important result highlights the role of LPC for HC in term of metabolic response. Indeed, in the case of a specific parietal brain activity (pointed out by increased LPC amplitude) the central topographic representation also becomes more active (deoxy-Hb decreasing as a marker of a supposed increased brain activity), as a form of focused activation which includes a larger brain network even for longer latencies (i.e., LPC) as described before for shorter latencies (i.e., N1).

This research opens interesting aspects linked to smell and 3D cross-modal representation. In fact, we can observe a common pathway where the brain areas act in terms of electrophysiological activation linked to metabolic activation/deactivation (Shin et al., 2018). This can help to understand the complex relationship of the brain dynamics in a cross-modal perceptive way with a hybrid system as EEG–fNIRS co-registration.

This study, however, highlights methodological limits due to the analysis not evaluating the entire EEG spectrum of alpha, beta, delta, gamma, and delta rhythms. This would have increased the complexity of the analysis, which, due to the small sample size, would have weakened the reliability of the data (Button et al., 2013). In fact, the evaluation of alpha/mu and delta rhythms, as mentioned above, has been chosen as the two rhythms seem to be mostly associated olfactory perception and cross-modal haptic stimulation in literature (Martin, 1998; McFarland et al., 2000; Freyer et al., 2012; Piarulli et al., 2018). Another limit is due to the exiguity of studies in literature that can guide the investigation of the hybrid EEG and fNIRS co-registration (Putze et al., 2014; Shin et al., 2018).

Although the analysis of the effects of the conditions does not give significant variations of rhythms, we can consider this aspect as a limit of a complete and simple pilot survey that allows us to explore various aspects, some strongly correlated with the task and others more related to general aspects of cortical and sensory non-specific activation, but through which common pathways are then highlighted within the hybrid co-registration. Even these limited aspects, especially in a pilot study, can be useful as a basis for structuring subsequent research studies (Amrhein et al., 2019; Haaf et al., 2019).

In fact, our results seem to be in line with cross-modal studies on cross-modal perception and shapes analysis (Lalanne and Lorenceau, 2004) where it is described that the perception follows from cross-modal processing, and that an integrated percept can emerge from unique representation that comes out of a common generalization when the stimuli are temporally and spatially connected to each other. This perceptive “communality” is highlighted by the same process modalities which must be carried out within aspects linked to dynamic network, where the predominant perceptive area can also include multisensory aspects when they are associated with each other during the stimuli perception (Spence, 2002; Lalanne and Lorenceau, 2004; Rubin et al., 2015).

## Data Availability Statement

The datasets generated for this study are available on request to the corresponding author.

## Ethics Statement

The studies involving human participants were reviewed and approved by the Ethics Committee of Psychology Department, Catholic University of Milan. The patients/participants provided their written informed consent to participate in this study.

## Author Contributions

SI developed the experimental design, the EEG and OERP data collection, and the EEG, OERP, and fNIRS data analysis. VC supported the technical aspects of the olfactometer. SI, RM, GF, and IV participated in the experimental phase, data analysis, and discussion. SI, MB, and GF wrote the manuscript. SI and MB conducted the supervision of the research, the planning and supervision of the experimental design, and data analysis.

## Conflict of Interest

The authors declare that the research was conducted in the absence of any commercial or financial relationships that could be construed as a potential conflict of interest.
